# *Tricholoma matsutake* may take more nitrogen in the organic form than other ectomycorrhizal fungi for its sporocarp development: the isotopic evidence

**DOI:** 10.1007/s00572-018-0870-8

**Published:** 2018-11-08

**Authors:** Lu-Min Vaario, Shambhu Prasad Sah, Mariko Norisada, Maki Narimatsu, Norihisa Matsushita

**Affiliations:** 1 Department of Forest Sciences, University of Helsinki, PO Box 27, FI-00014 Helsinki, Finland; 2 Asian Natural Environmental Science Center, The University of Tokyo, 1-1-1 Yayoi, Bunkyo-ku, Tokyo, Japan; 3 Iwate Prefectural Forestry Technology Center, 560-11 Kemuyama, Yahaba, Iwate Japan; 4 Graduate School of Agricultural and Life Sciences, The University of Tokyo, 1-1-1 Yayoi, Bunkyo-ku, Tokyo, Japan

**Keywords:** Stable isotope, Ectomycorrhizal fungi, Source, Sporocarps, *Tricholoma matsutake*

## Abstract

*Tricholoma matsutake* is an ectomycorrhizal (ECM) fungus capable of in vitro saprotrophic growth, but the sources of C and N used to generate sporocarps in vivo are not well understood. We examined natural abundance isotope data to investigate this phenomenon. For this purpose, C, N and their stable isotopes (^13^C, ^15^N) content of fungal sporocarps and their potential nutrient sources (i.e., foliage, litter, fine roots, wood, and soil) were investigated from two well-studied sites in Finland and Japan. Our results show that δ^13^C values of *T. matsutake* and other fungal groups are consistent with those of most studies, but a very high δ^15^N value (16.8‰ ± 2.3) is observed in *T. matsutake*. Such isotopic pattern of fungal δ^15^N suggests that matsutake has a greater proteolytic potential to digest chemically complex ^15^N-enriched organic matter and hydrophobic hyphae. This assumption is further supported by a significant and positive correlation between δ^13^C_cap–stipe_ and δ^15^N_cap–stipe_ exclusively in *T. matsutake*, which suggests common C and N sources (protein) possible for isotopically enriched cap. The ^13^C increase of caps relative to stipe presumably reflects greater contents of ^13^C-enriched protein than ^13^C-depleted chitin. We conclude that *T. matsutake* is a typical ECM fungus which obtains for its sporocarp development for both C and N from a common protein source (vs. photosynthetic carbon) present in soil organic matter.

**Electronic supplementary material:**

The online version of this article (10.1007/s00572-018-0870-8) contains supplementary material, which is available to authorized users.

## Introduction

*Tricholoma matsutake* (S. Ito & S. Imai) Singer is an ectomycorrhizal (ECM) fungus that forms a symbiotic association with pine and spruce trees in Asia and northern Europe (Ogawa 1978; Yamada et al. 1999; Vaario et al. 2010). Matsutake is one of the most expensive edible mushrooms available (Hall et al. 2003). While many investigators have focused on optimal growth conditions and the artificial culture of this high-value fungus (Kawai and Ogawa 1981; Lee et al. 1984; Guerin-Laguette et al. 2005; Yamada et al. 2006), artificial cultivation remains difficult and unsatisfactory (Wang et al. 2012). Earlier studies of the ecological interactions between *T. matsutake* and other soil microbes with the host plant as well as studies of edaphic qualities and climate (see review, Vaario et al. 2017) have tried to identify the factors that regulate sporocarp formation. One aspect that has yet to be investigated thoroughly in this respect concerns the physiology of matsutake nutrition.

Matsutake behaves as a typical ECM fungus in laboratory and field experiments (Yamada et al. 1999; Gill et al. 2000), in that it associates with host plants to form a mutualistic symbiotic relationship. In addition to its symbiotic behavior, matsutake is also known to produce a range of extracellular enzymes including amylases, cellulases and proteinases (Terashita et al. 1995), and β-glucosidase (Vaario et al. 2002; Kusuda et al. 2006) in liquid culture and xylosidase in bark fragments (Vaario et al. 2012). *Tricholoma matsutake* was shown to utilize pine bark, spruce, and birch hemicellulose as the principal carbon source in vitro (Vaario et al. 2002, 2012). However, the extent to which facultative saprotrophy is an adaptation to nutrient stress or an essential feature of vegetative mycelium growth and further sporocarp formation in nature is not yet clear.

Stable isotope analysis has proved to be a useful tool for understanding the trophic niche of different fungi in natural situations because isotopic variation in plants, soil, and fungi is determined by the mechanisms of resource acquisition, loss, and internal cycling (Hobbie et al. 1999; Hobbie et al. 2001). Natural stable isotope (^15^N and ^13^C) content can effectively identify nutritional strategies in fungi (Hobbie et al. 2001; Mayor et al. 2009). Previous studies have shown that isotopic values of sporocarps were similar to hyphae, and therefore useful in the study of belowground C and N dynamics (Högberg et al. 1999; Taylor et al. 1997). Understanding the variation in ^15^N and ^13^C abundance among matsutake sporocarps and sympatric ECM and SAP (saprotrophic) fungal sporocarps from the same sites could shed some light on matsutake nutritional physiology. In addition, isotopic patterns in different fungal components may provide some insight into mechanisms creating isotopic differences among fungi (Taylor et al. 1997).

We selected two sites in Finland and Japan where the occurrence of *T. matsutake* was established and well studied (Lian et al. 2006; Narimatsu et al. 2015; Vaario et al. 2011, 2015). In this study, we measured the natural abundance of ^13^C and ^15^N to elucidate the nutrient supply source(s) of matsutake and aimed (1) to compare the isotopic patterns in *T. matsutake* with other ECM and SAP sporocarps to ecosystem components within the sites and (2) to compare the variation of %C, %N, δ^13^C, and δ^15^N of sporocarps between caps and stipes to understand the functional attributes of matsutake in nature. The aim of this study was to examine whether the fungal isotopic pattern could provide any new insight of the ecological role of *T. matsutake* species in nature.

## Methods and materials

### Study sites and sampling

This study was conducted in two study sites; one located in Nuuksio National Park in southern Finland (SF: 60° 18′ N, 24° 31′ E) and the other a prefectural forest in northeast Japan (NJ: 39° 56′ N, 141° 14′ E). A relative sandy forest soil in SF site (Vaario et al. 2012) and brown forest soil in NJ site (Narimatsu et al. 2015). The occurrence of *T. matsutake* has been monitored for nearly 10 years at SF (Vaario et al. 2015) and for 23 years at NJ (Narimatsu et al. 2015). No management activities, such as thinning, cutting, or burning, were conducted at these sites during the study period.

Sporocarps of *T. matsutake* and other macrofungi were separately collected in the study sites during the fruiting season in 2013 (SF) and 2016 (NJ). Based on long-term field observations, there are several matsutake fruiting patches in both locations. In this study, five patches at SF and three patches at NJ were included for sampling. One matsutake sporocarp spot in each patch was randomly selected as the location from which environmental samples were collected. A soil corer (inner diameter, 50 mm) was driven to 10-cm depth at five locations at SF, and three at NJ. Soil samples were parsed into organic (OS) and mineral (MS) soil fractions, living fine roots (FR: < 2-mm diameter size) were removed from the mineral soil. FR were picked under a dissecting microscope and washed with sterile water to remove soil particles from the surface. Litter material (LI) and newly fallen foliage material (FO), about 100 ml, were collected in the same location in each patch. The nearest trees to the sampling locations were *Pinus sylvestris* L. at SF, and *Pinus densiflora* Siebold & Zucc. at NJ. Wood (WO) was sampled just beneath the bark at about 1.5 m from the ground. The sampled sporocarps were apparently healthy and separated into cap and stipe. Only inner tissue of cap or stipe was sampled for further analysis. Some sporocarps were too small for accurate dry-weight analysis, so the cap and stipe tissues were pooled for the analysis. Such samples were excluded from the calculation of fractionation between cap and stipe. All samples were dried at 50 °C overnight and then ground in a Mixer Mill MM400 (Retsch, Germany) for at least 2 min. Powdered samples were stored in air-tight glass containers at room temperature prior to stable isotope analysis.

### Identification of sporocarps

All sporocarps collected in the study site were identified to species according to morphology (Imazeki and Hongo 1987, 1989; Salo et al. 2006) and confirmed with nucleotide sequences (Table S1). Genomic DNA was extracted from 0.25 g of sporocarp tissue with the NucleoSpin Plant II (Macherey-Nagel) for SF samples and PrepMan Ultra Sample Preparation Reagent (Thermo Fisher Scientific Inc.) for NJ samples according to the manufacturer’s instructions. The internal transcribed spacer (ITS) region of the rDNA was amplified with ITS1F (5′-CTT GGT CAT TTA GAG GAA GTA A-3′) (Gardes and Bruns 1993) and ITS4 primers (5′-TCC TCC GCT TAT TGA TAT GC-3′) (White et al. 1990). PCR amplification was performed with Biotools polymerase (B & M Laboratories, Madrid, Spain) or KAPA Taq Extra PCR Kit (Kapa Biosystems, Wilmington, MA) with the following thermal profile: initial denaturation for 8 min at 95 °C; 35 cycles of denaturation for 1 min at 95 °C, annealing for 1 min at 58 °C, and extension for 1 min at 72 °C; and a final extension step of 7 min at 72 °C. PCR products were sequenced by a commercial sequencing service (Macrogen Inc.) with the same primers used in amplification. Sequences were aligned with those available in GenBank using the BLAST algorithm and deposited under the accession numbers KM517228 to KM517248 for samples from SF site, LC373239 to LC37325 for the samples from NJ site.

### Chemical and isotopic analysis of soil, plant, and sporocarp samples

The C and N concentrations of plant, soil, and sporocarp samples were determined using a CN element analyzer (Elementar Analysensysteme GmbH Germany) using direct combustion at 850 to 1150 °C. Samples of 3 mg or 15 mg (mineral soil) were combusted and C and N isotope ratios were measured on a Finnigan MAT Delta plus stable isotopic ratio mass spectrometer (IRMS) equipped with an elemental analyzer (SF samples) or a Delta V Advantage equipped with an elemental analyzer (Thermo Fisher Scientific, Bremen, Germany) (NJ samples). Results of the IRMS measurement were given in δ notation. The δ values of C and N isotopes are expressed as follows:

δ^13^C or δ^15^N (‰) = (*R*_sample_/*R*_standard_ − 1) × 1000, where, *R*_sample_ = ^13^C/^12^C or ^15^N/^14^N in samples; *R*_standard_ = ^13^C/^12^C or ^15^N/^14^N present in a standard. Pee Dee Belemnite for C and air for N was used as the standard for all samples.

Samples from two sites were measured separately at the Center for Stable Isotope Research and Analysis, University of Göttingen, Germany (SF samples) and the University of Tokyo (NJ samples). As an error check, five SF samples were analyzed with the system in Japan and noted only trivial differences in the data obtained.

### Isotopic patterns in different fungal components

The isotopic difference from stipe to cap was calculated as δ^13^C_(cap–stipe)_ = δ^13^C_cap_ − δ^13^C_stipe_. We compare the difference between cap and stipe in *T. matsutake*, other ECM fungi, and SAP fungi.

### Statistical analysis

Stable isotope values of sporocarps and sources are presented ± standard deviation (SD) in tables and in figures. All datasets were tested separately for exhibiting normality and homogeneity of variance. Mean values for the forest components were compared using a one-way ANOVA followed by the Tukey post hoc test, *α* = 0.05. The non-parametric Kruskal–Wallis test was used when assumptions of normality or equality of variance were not met. A student’s *t* test was applied to compare the values between cap and stipe. A Pearson correlation was employed to evaluate the relationship between isotopic enrichment and C and N enrichment in caps vs. stipes in *T. matsutake*, other ECM fungi, and SAP fungi. All statistical analyses were performed with SPSS (version 20.0; SPSS Inc., Chicago, Illinois).

## Results

### Sporocarps fruiting in the study sites

Matsutake fruited continuously at SF during the observation period from August 22, 2013 to September 15, 2013. During the same period, 32 sporocarps of other macro fungi were found, including 25 ECM fungi belonging to genera such as *Amanita*, *Boletus*, *Cortinarius*, *Hydnum*, *Leccinum*, *Russula*, and *Suillus*, and 7 sporocarps of SAP fungi belonging to *Armillaria* and *Hygrophoropsis*. The peak matsutake fruiting time was in the beginning of September, with other macrofungal species peaking 1–3 weeks later.

Similarly, *T. matsutake* fruited continuously at NJ during the observation period from September 23, 2016 to November 14, 2016. During the same period, sporocarps of other macrofungal species were found, including ECM fungi belonging to nine genera (e.g., *Cantharellus*, *Cortinarius*, *Entoloma*, *Lactarius*, *Lyophyllum*, *Phellodon*, *Russula*, *Sarcodon*, and other *Tricholoma*), and saprophytes belonging to *Mycena* and *Rhodocollybia*. The peak matsutake fruiting time was in the beginning of October.

### C, N, and their isotopes (^13^C and ^15^N) along an environmental gradient from living tree to soil and in sporocarps

**Fig. 1:**
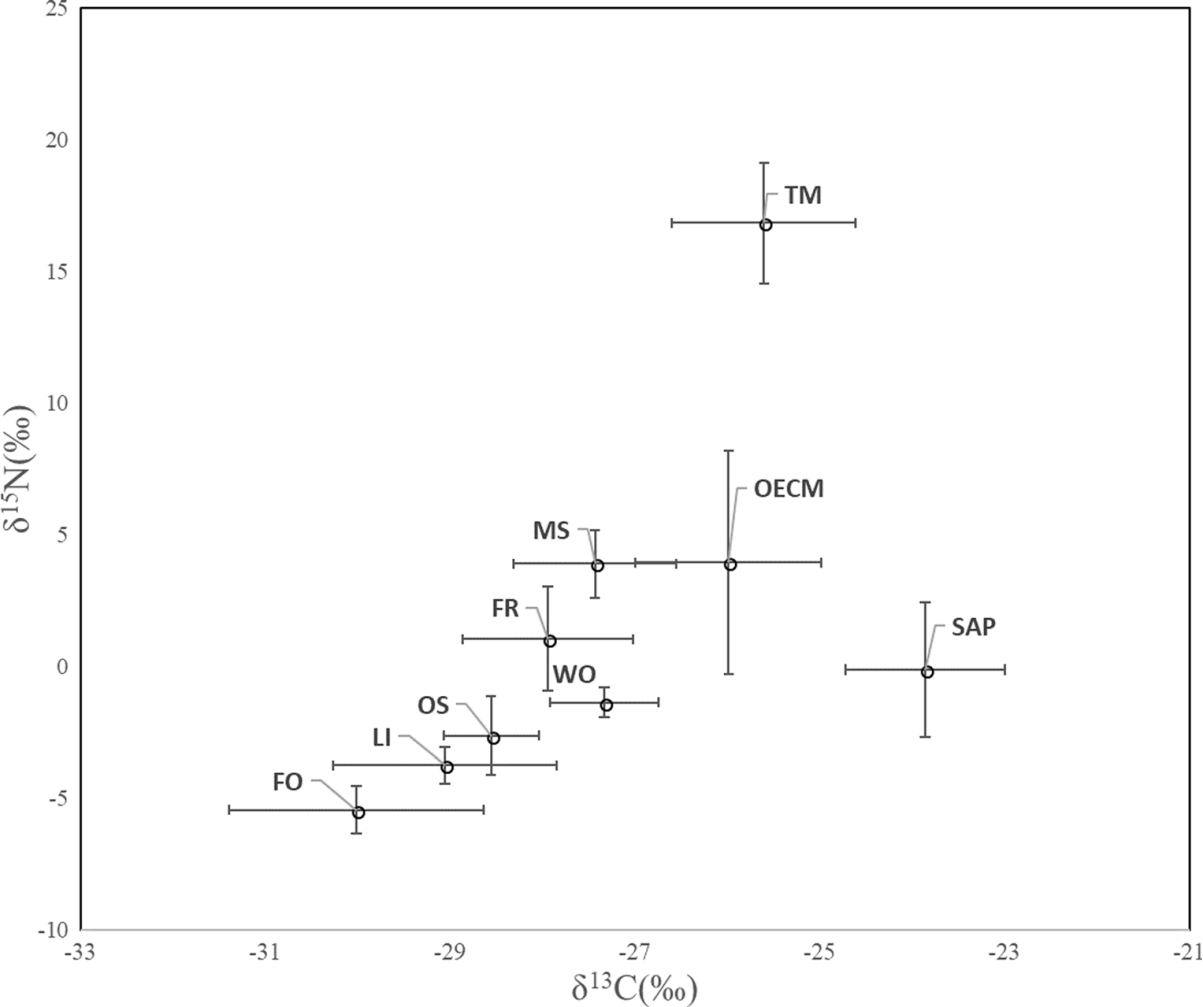
Carbon and nitrogen stable isotope values for different forest components and sporocarps of the study sites. Data points represent means with standard deviation. TM, *Tricholoma matsutake*; OECM, other ECM fungi; SAP, saprotrophic fungi; MS, mineral soil; FR, fine root; OS, organic soil; WO, wood; LI, litter; FO, foliage

The δ^13^C and δ^15^N values of the potential nutrient sources (the plant and soil material) from both sites increased from aboveground to belowground with depth increment except for WO (Fig. 1; Table 1). C concentration among the potential nutrient sources showed that the lowest value was in the mineral soil samples, and highest in litter samples, while %N differed significantly among the source samples (i.e., fine root, organic soil, foliage > litter > mineral soil. Mineral soil was significantly poor in N (Table 1).

**Table 1:** Mean values of carbon and nitrogen stable isotope values and %C and %N of different forest compartments in this study

Sample type	Sample number	δ^13^C (‰)	δ^15^N (‰)	C%	N%
Foliage (FO)	7	− 30.02 (1.38)^d^	− 5.42 (0.89)^d^	47.74 (3.65)^ab^	1.15 (0.40)^a^
Litter (LI)	13	− 29.06 (1.21)^cd^	− 3.73 (0.69)^cd^	51.32 (2.61)^a^	0.60 (0.12)^b^
Wood (WO)	7	− 26.48 (0.59)^a^	− 2.79 (0.58)^c^	46.30 (3.61)^ab^	0.09 (0.16)^c^
Organic soil (OS)	9	*− 28.56 (0.52)* ^bcd^	− 2.62 (1.49)^c^	43.39 (9.99)^ab^	1.34 (0.47)^a^
Fine root (FR)	7	− 27.94 (0.92)^abc^	1.05 (1.98)^b^	42.03 (8.80)^b^	1.03 (0.15)^a^
Mineral soil (MS)	8	− 27.43 (0.88)^ab^	3.90 (1.27)^a^	3.77 (1.65)^c^	0.12 (0.04)^c^

*The mean values (± SD) within forest compartments (source pools) were compared using a one-way ANOVA followed by the Tukey post hoc test; the same letter indicates no significant different, *α* = 0.05

**Fig. 2:**
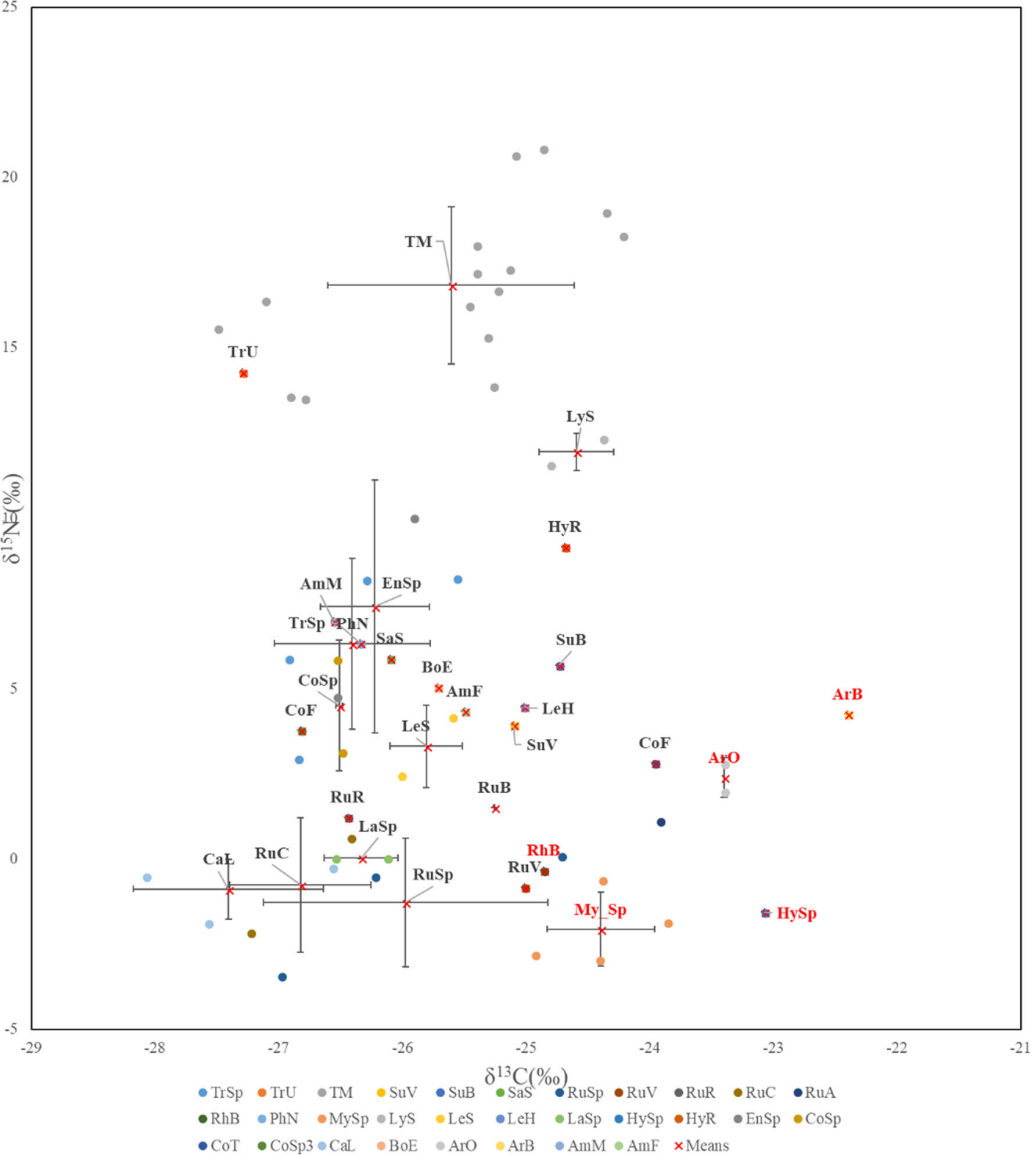
Carbon and nitrogen stable isotope values for all sporocarps in species level. TrSp, *Tricholoma* sp.; TrU, *T. ustela*; TM, *T. matsutake*; SuV, *Suillus variegatus*; SuB, *S. bovinus*; SaS, *Sarcodon scabrosus*; RuSp, *Russula* sp.; RuV, *R. vinosa*; RuR, *R. rhodopoda*; RuC, *R. claroflava*; RuA, *R. amethystine*; RhB, *Rhodocollybia butyracea*; PhN, *Phellodon niger*; MySp, *Mycena s*p.: LyS, *Lyophyllum semitale*; LeS, *Leccinum scabrum*; LeH, *L. holopus*; LaSp, *Lactarius* sp.; HySp, *Hygrophoropsis* sp.; HyR, *Hydnum repandum*; EnSp, *Entoloma* sp.; CoSp, *Cortinarius* sp.; CoT, *C. turgidus*; CoF, *C. fulvescens*; CaL, *Cantharellus luteocomus*; BoE, *Boletus edulis*; ArO, *Armillaria ostoyae*; ArB, *A. borealis*; AmM, *Amanita muscaria*; AmF, *A. fulva*. Means ± SD

Compared to the different forest compartments, the δ^13^C and δ^15^N values of *T. matsutake* clustered independently did not overlap with other fungal species and forest compartments, except for the δ^13^C values of *T. matsutake* which were similar to the other ECM fungi (Figs. 1 and 2).

Among all tested sporocarps, the δ^13^C and δ^15^N values of sporocarps greatly varied in species level. δ^13^C values ranged from − 22.4 (‰) in *Armillaria borealis*, a saprotrophic fungus to − 28.1 (‰) in *Cantharellus luteocomus*, an ectomycorrhizal fungus; δ^15^N values ranged from 20.9 (‰) in *T. matsutake* to − 3.4 (‰) in *Russula sp*. (Fig. 2). Among all sporocarps of *T. matsutake*, means of δ^13^C and δ^15^N values were − 25.6‰ ± 0.99 and 16.8‰ ± 2.3. Only one species, *T. ustale*, located within the matsutake cluster (Fig. 2).

### Isotopic difference in caps versus stipes

The isotopic difference from stipe to cap was typical for sporocarps but varied significantly among matsutake and other ECM fungi, but not in SAP fungi. δ^13^C_cap–stipe_ was significantly higher in matsutake relative to other ECM fungi. However, we did not find significant differences with respect to enrichment of δ^15^N_cap–stipe_ among the three groups, or %C_cap–stipe_ and %N_cap–stipe_ (Table 2).

**Table 2:** The difference in cap vs. stipes for carbon and nitrogen stable isotope values

Sporocarps	Sample number	δ^13^C (‰)			δ^15^N (‰)			C%			N%		
		Cap	Stipe	Cap–stipe**	Cap	Stipe	Cap–stipe	Cap	Stipe	Cap–stipe	Cap	Stipe	Cap–stipe
OECM	38	− 25.68*	− 26.26	0.55^b^	5.26*	2.51	2.73	41.90	41.00	0.77	4.33*	2.73	1.37
		1.07	1.10	0.87	4.55	4.17	2.33	2.30	2.48	2.49	1.51	1.11	0.25
TM	15	− 25.02*	− 26.34	1.25^a^	18.31*	15.15	3.08	40.38*	38.88	1.80	3.93*	2.03	1.82
		1.09	0.85	0.75	2.66	1.97	1.63	2.88	3.59	5.23	1.39	1.01	1.23
SAP	9	− 23.58	− 24.44	0.98 ^ab^	0.71	− 1.45	3.21	40.64	40.57	− 0.10	5.93*	3.80	1.73
		0.79	1.14	0.80	2.98	2.27	2.46	3.82	1.23	4.75	1.36	1.25	0.76

*The statistical difference of mean values (SD in every second row) between cap and stipe is marked with asterisks with examined by student *t* test, *α* = 0.05

**The mean values (SD in every second row) of cap–stipe within three fungal groups were compared using a one-way ANOVA followed by the Tukey post hoc test; the same letter indicates no significant different, *α* = 0.05

δ^13^C_cap–stipe_ and δ^15^N_cap–stipe_ were significantly and positively correlated only in matsutake (Pearson correlation coefficient was 0.82, *p* = 0.01), but not significantly in other ECM or SAP fungi. %N_cap–stipe_ was significantly and positively correlated with δ^13^C_cap–stipe_ in all three groups fungi (0.85, *p =* 0.01 in TM; 0.52, *p =* 0.01 in OECM; 0.89, *p =* 0.01 in SAP).

## Discussion

To our knowledge, this is the first in situ study of the natural abundance of C and N isotopes in sporocarps of *T. matsutake*. Overall, our data showed a trend of increasing of δ^13^C and δ^15^N in the different forest compartments from foliage, litter to deeper soil horizons, in consistent with those found previously for boreal forests elsewhere (Taylor et al. 1997; Zeller et al. 2007; Hobbie et al. 2012) based on samples collected from Finland and Japan. We suggested the following new findings: (1) the sporocarps of *T. matsutake* comprised of similar range of δ^13^C value as in other ECM fungi; (2) the sporocarps of *T. matsutake* showed very high δ^15^N values in comparison to other ECM and SAP fungi, suggesting that matsutake may obtain N from chemically complex ^15^N-enriched organic matter and have proteolytic capabilities, adapted to N-limited condition; 3) a significant and positive correlation between δ^13^C_cap–stipe_ and δ^15^N_cap–stipe_ existed in *T. matsutake*, suggesting more efficient nutrient uptake from soil organic matter in matsutake.

Regarding the isotopic pattern in δ^13^C for *T. matsutake*, the results presented here are in agreement with those of most studies, where δ^13^C separates nearly all ECM and SAP fungi into two separate clusters (Hobbie et al. 1999; Kohzu et al. 1999). Such results indicate the different kinds of resources utilization pattern and ecological role in all these fungal types. Up to date, most of the studies showing the saprotrophic potential of *T. matsutake* (Terashita et al. 1995; Hur et al. 2001; Kusuda et al. 2006; Vaario et al. 2002) are limited to laboratory conditions and investigations in situ are scarce. Though we did not observe high δ^13^C values in *T. matsutake* sporocarps, the significant positive correlation between δ^13^C_cap–stipe_ and δ^15^N_cap–stipe_, observed only in *T. matsutake*, may indicate a common C and N source (protein, see details below) in this species. However, further study on the isotopic analysis of compound specific chemical components of fungal sporocarps and their substrates are further needed to testify our hypothesis.

In contrast to δ^13^C, δ^15^N isotope values of *T. matsutake* in the present study exhibited a very high value in comparison to other ECM fungi (except for *T. ustale* in this study). Such kind of higher values of ^15^N in matsutake has also been observed in the American matsutake (*Tricholoma magnivelare*) in northwestern USA (Trudell et al. 2003). We propose the following potential mechanisms for such ^15^N-enriched sporocarps in matsutake:

Firstly, soil depth at which taxa obtain their N could explain for their δ^15^N values. In general, soil δ^15^N increase with their increasing depth from surface layer to mineral layer in the range from 1.6 to 5‰ (see review by Hobbie and Högberg 2012). In our study, we have observed even higher range (7.6 ‰) of δ ^15^N values from litter (− 3.7 ‰) to mineral soil (+ 3.9 ‰) layer. The *T. matsutake* mycorrhizal association is mostly restricted to the B-layer mineral soil, which is the most visible whitish mycelium–soil aggregated zone (Yamada et al. 2006). Because the observed value of ^15^N in matsutake is far higher than the usual ^15^N values in mineral soil, we hypothesize that mineral soil N uptake plays only a minor role in ^15^N enrichment of matsutake. However, a modeled value of total ^15^N has been proposed for fractionation against ^15^N (8–10‰) during internal transfer of N from ECM fungi to tree foliage and 3‰ during formation of fungal fruiting bodies (Hobbie et al. 2000; Hobbie and Colpaert 2003; Hobbie et al. 2005). If we consider this value in mass balance calculation, such ^15^N enrichment in matsutake seems to be feasible, but a further investigation is needed.

Secondly, the high δ^15^N value in matsutake is an indicator of organic N and NH_4__N uptake from soil. The great variation of ^15^N content among ECM taxa has been reported to be related with organic N utilization (Taylor et al. 1997). The literature study shows that mycorrhizal taxa with proteolytic capabilities generally have high δ^15^N values (Lilleskov et al. 2002). Enzymatic activities of proteases from the mycelia of *T. matsutake* were reported previously (Terashita and Kono 1989). Kawai and Abe (1976) reported that dried beer yeast, corn steep liquor, casein hydrolysate, and polypeptone were good N source for matsutake mycelium culture, but not nitrate. In addition, Koba et al. (2003) reported higher δ^15^N in NH_4_-N in the mineral soil ca. 5–6 ‰ in average in contrast to lower values of NO_3_-N. However, soil NH_4_-N is only slightly ^15^N enriched. Therefore, the contribution of NH_4_-N to the higher δ^15^N (+ 16.8‰ in average) in *T. matsutake* is probably low.

The massive carbonized root tips colonized by matsutake hyphae can be usually found in matsutake shiro (a dense mat of fungal hyphae formed in association with pine roots and soil particles (Hosford et al. 1997)) soil (Gill et al. 2000; Yamada et al. 2006). Such dead plant–fungal material could be considered as the organic N source existing in matsutake shiro. In addition, we observed a relatively higher content of organic matter and %N in the highly matsutake producing spots than that in non-matsutake spots in our previous study, although root material was excluded from the soil analysis (Vaario et al. 2012). Thus, it seems possible for matsutake to gain the majority of its N in the organic form (amino acids and proteins) from the soil which is greatly ^15^N enriched.

Thirdly, functional attributes may correlate with N isotope pattern in ECM fungi (Hobbie and Högberg 2012). The link between rhizomorph abundance and δ^15^N was suggested (Lilleskov et al. 2002; Trudell et al. 2004), especially with how they explore the soil and with the hydrophobicity of ectomycorrhizas (Agerer and Raidl 2004), which hydrophobic hyphae mean that exploring hyphae could create mycelial patches at water-air interface of numbers of substrates. Macrofungi with high biomass usually sequester N in extra-radical hyphae and rhizomorphs (Hobbie and Agerer 2010). However, *T. matsutake*, as other *Tricholoma* species, does not belong to the category of ECM fungi with long-distance transport mycelia. Matsutake has unique features in its mycorrhiza, e.g., a thin and undifferentiated fungal sheath and carbonized root tips that resemble general plant necrotic reaction (Yamada et al. 2001; Yamada et al. 2006), but *T. matsutake* has hydrophobic hyphae (Guerin-Laguette et al. 2003), which could explain the high level of ^15^N enrichment in matsutake sporocarps.

Isotopic difference in caps versus stipes was observed in this study. Previous studies have indicated that isotopic patterns in different fungal components may be influenced by the elemental composition in the fungal tissues. Taylor et al. (1997) concluded that higher %N and δ^15^N in cap than in stipes greater ^15^N-enriched protein and less ^15^N-depleted chitin in caps than in stipes. Although the distribution of proteins within a sporocarp and changes in protein content during the development of a sporocarp remain mostly unclear. Vetter and Rimóczi (1993) reported crude protein contents were 36.4% and 11.8% in cap and stipe at the sporocarp cap 5- to 8-cm stage in cultivated *Pleurotus ostreatus*, respectively. Therefore, Hobbie et al. (2012) suggested that a constant chitin content between caps and stipes is a reasonable simplification, with large increase in protein content from stipes to caps driving changes in %N and δ^15^N. The differences of ^13^C and ^15^N content between caps and stipes was not observed in SAP fungi in this study. The relative small size of sporocarps of SAP fungi in this study can obscure the difference between cap and stipe (Trudell et al. 2004).

The significant positive correlation between δ^13^C_cap–stipe_ and δ^15^N_cap–stipe_ (*p* < 0.01) was observed only in *T. matsutake* (not in other fungal types like OECM and SAP) in this study. Although there were more sample numbers in *T. matsutake* sporocarps than other species, such result might still reflect common C and N sources (protein) for isotopically enriched cap relative to the stipes; ^13^C increase of caps relative to stipe presumably reflects greater contents of ^13^C-enriched protein than ^13^C-depleted chitin and carbohydrates (Webb et al. 1998). Taylor et al. (1997) reported that protein and amino acids were about 10‰ enriched in ^15^N relative to chitin in fungi. These authors also reported higher ^13^C and ^15^N abundance and %N in caps relative to stipes, which is attributed to the presence of more ^15^N- and ^13^C-enriched protein and amino acids in caps than in stipes. N contents affect the ^13^C contents of sporocarp, as sporocarp N is protein and hence fungal protein is ^13^C enriched compared to chitin. Hence, we presume that the higher the protein (organic C) in the sporocarp tissue, the higher will be the ^13^C and %N values of the sporocarp. The isotopic patterns in different fungal components may provide some insights into fungal nutrition mechanisms creating differences among fungi; however, the distribution and changes of proteins and chitin within a sporocarp during the development of a sporocarp is unclear and remain to be further explored.

In conclusion, dual isotopic analysis (^13^C and ^15^N) of fungal sporocarps and their bulk substrates, in general, functions as ecological indicator of the C and N uptake in fungal species. The isotopic values suggest that matsutake, a typical ectomycorrhizal fungus, may have common source of C and N uptake from soil organic matter (protein as common source); whereby, we assume that it obtains most of its N in the form of organic N (^15^N enriched). However, further researches on the compound specific isotopic analysis of sugar, protein, and chitin in fungal sporocarps and ecosystem pools are required to address these issues in more details. This study can be useful for challenging the matsutake cultivation in both forest management and in nursery.

## Electronic supplementary material

ESM 1 (DOCX 26 kb)
