# Supplementary material for: Tricholoma matsutake may take more nitrogen in the organic form than other ectomycorrhizal fungi for its sporocarp development: the isotopic evidence
Source: Mycorrhiza. 2018 Nov 8;29(1):51–9. doi: 10.1007/s00572-018-0870-8 (PMC6311186; doi:10.1007/s00572-018-0870-8)
Supplement: Supplementary file 1 — (DOCX 26 kb) [file 572_2018_870_MOESM1_ESM.docx]

Supplement Table 1. A list of other macrofungal species found in the study sites

*Excluded in the analysis
